# Nrf2, a PPARγ Alternative Pathway to Promote CD36 Expression on Inflammatory Macrophages: Implication for Malaria

**DOI:** 10.1371/journal.ppat.1002254

**Published:** 2011-09-15

**Authors:** David Olagnier, Rose-Anne Lavergne, Etienne Meunier, Lise Lefèvre, Christophe Dardenne, Agnès Aubouy, Françoise Benoit-Vical, Bernhard Ryffel, Agnès Coste, Antoine Berry, Bernard Pipy

**Affiliations:** 1 Université de Toulouse, UPS, UMR-MD3, Relations Hôte-Parasites Polarisation des Macrophages et Récepteurs Nucléaires dans les Pathologies Inflammatoires et Infectieuses, Toulouse, France; 2 Université de Toulouse, UPS, UMR 152 IRD, Pharma-Dev, Toulouse, France; 3 UPR 8241CNRS, Laboratoire de Chimie de Coordination, Toulouse, France; 4 Université Orléans, UMR 6218 CNRS, Laboratoire d'immunologie et d'embryologie moléculaire, Orléans, France; Faculdade de Medicina da Universidade de Lisboa, Portugal

## Abstract

CD36 is the major receptor mediating nonopsonic phagocytosis of *Plasmodium falciparum*-parasitized erythrocytes by macrophages. Its expression on macrophages is mainly controlled by the nuclear receptor PPARγ. Here, we demonstrate that inflammatory processes negatively regulate CD36 expression on human and murine macrophages, and hence decrease *Plasmodium* clearance directly favoring the worsening of malaria infection. This CD36 downregulation in inflammatory conditions is associated with a failure in the expression and activation of PPARγ. Interestingly, using siRNA mediating knock down of Nrf2 in macrophages or Nrf2- and PPARγ-deficient macrophages, we establish that in inflammatory conditions, the Nrf2 transcription factor controls CD36 expression independently of PPARγ. In these conditions, Nrf2 activators, but not PPARγ ligands, enhance CD36 expression and CD36-mediated *Plasmodium* phagocytosis. These results were confirmed in human macrophages and *in vivo* where only Nrf2 activators improve the outcome of severe malaria. Collectively, this report highlights that the Nrf2 transcription factor could be an alternative target to PPARγ in the control of severe malaria through parasite clearance.

## Author Summary

Severe and fatal malaria is still increasing both in incidence and in its resistance to antimalarial agents. The improved understanding of immune mechanisms mediating *Plasmodium* elimination might therefore offer a complementary way to conventional therapeutic interventions. The main host innate immune defense mechanism against *Plasmodium falciparum* is the engulfment by macrophages through CD36, the macrophage receptor recognizing infected erythrocytes. The up-regulation of CD36 on macrophages therefore represents an alternative way to favor parasite clearance during infection. Severe malaria infection is associated with an excessive production of proinflammatory markers and an inability to control parasite proliferation. We demonstrate here that malaria-induced inflammation down regulates CD36 expression on macrophages and favors the worsening of malaria infection. The conventional way to promote CD36 expression through PPARγ nuclear receptor is inefficient under malaria inflammatory processes. Interestingly, we establish that the Nrf2 transcription factor may substitute PPARγ to promote CD36 expression and its associated functions in inflammatory conditions. As a consequence, only Nrf2 but not PPARγ activators improve the outcome of severe malaria *in vivo*. This paper which highlights a new area of application for Nrf2 activators in infectious diseases, heralds the emergence of a new therapeutic strategy against severe malaria.

## Introduction

Mononuclear phagocytes represent the first line of innate immune defense against pathogens through mechanisms involving recognition by pattern-recognition receptors (PRRs) of highly structurally conserved microbial structures, known as pathogen-associated molecular patterns [1]. Among the PRRs family, the class B scavenger receptor CD36, initially known as a receptor for the uptake of oxidatively low density lipoprotein, also mediates the recognition and the elimination of apoptotic cells and bacteria [2], [3]. Additionally, the CD36 receptor specifically recognizes *Plasmodium falciparum* parasitized-erythrocytes (*Pf*PEs), resulting in a CD36-dependent nonopsonic phagocytosis of *Pf*PEs and a decrease in parasite-induced TNF-α secretion [4]. Consistently, CD36-deficient macrophages displayed a marked phagocytic defect for parasitized erythrocytes compared with wild-type macrophages [5]. Furthermore, a recent study demonstrates *in vivo* the importance of CD36 receptor expression on macrophages during malaria infection. Indeed, CD36^−/−^ mice present a defect in parasite clearance [5].

CD36 expression is under the transcriptional control of a PPARγ nuclear receptor. As a consequence, PPARγ ligands, such as thiazolidinediones, or IL4 and IL13 Th2 cytokines, promote CD36 expression on macrophages [6]–[8]. Moreover, rosiglitazone and IL13 have been shown to promote *in vitro* an increase in CD36-mediated phagocytosis and a decrease in malaria parasite-induced TNF-α release both on murine macrophage and human monocytes [4], [8], [9]. More recently, rosiglitazone treatment has been shown *in vivo* to reduce parasitemia level in the *Plasmodium chabaudi chabaudi* AS murine experimental model through the CD36 pathway [9]. Pharmacological modulation of CD36 expression on macrophages might therefore contribute to enhance parasite elimination and limit host inflammatory deleterious response to malaria infection.

Nevertheless, much of the pathology associated with malaria infection is a result of excessive and uncontrolled production of proinflammatory markers and cytokines [10], [11]. In this acute malaria inflammatory context, we previously demonstrated that CD36 receptor expression was reduced on the surface of circulating monocytes from *P. falciparum* infected patients [12]. In line with this, Th1 cytokines, such as TNF-α and IFNγ decrease CD36 expression both on monocytes and macrophages [13], [14]. Interestingly, this CD36 downregulation was correlated with a marked reduction in PPARγ activation upon TNF-α stimulation [13]. Collectively, all these data suggest that inflammatory processes might negatively regulate PPARγ expression and activation in macrophages.

Surprisingly, PPARγ^−/−^ macrophages did not present a totally abolished CD36 phenotype [8], [15]. This data suggests the existence of alternative pathways controlling CD36 expression on macrophages. In this study, we focused on NF-E2 related factor 2 (Nrf2), a transcription factor involved in the prevention of severe inflammatory diseases [16], that is activated in response to oxidative stress and electrophiles agents, such as sulforaphane or diethylmaleate. We previously demonstrated that the anti-TNFα antibody treatment increased CD36 expression on human monocytes through the enhancement of reactive oxygen species production independently of PPARγ [13]. Nrf2 was also shown to play an important role in the regulation of CD36 expression [17]–[19]. We therefore postulated that Nrf2 transcription factor might substitute PPARγ to promote CD36 expression and hence CD36-mediated phagocytosis of *Pf*PEs during acute inflammatory processes.

In this study, we show *in vitro* on murine and human monocytes-derived macrophages (hMDMs) and *in vivo* in murine inflammatory-induced severe malaria model, that inflammatory processes downregulate CD36 expression and CD36-mediated *Plasmodium* clearance, exacerbating the development of severe malaria infection. In acute inflammatory conditions, PPARγ ligands were unable to promote CD36 expression and subsequently to restore the loss of CD36-mediated *Plasmodium* clearance. Interestingly, we demonstrate the existence of an alternative pathway controlling CD36 expression in inflammatory conditions independently of PPARγ both on murine and human inflammatory macrophages. We established *in vitro* and *in vivo* that the Nrf2 transcription factor is essential to promote CD36 expression and *Plasmodium* clearance and therefore control malaria infection. This report highlights that Nrf2 transcription factor could be a therapeutic target in the control of severe malaria infection.

## Results

### Inflammatory conditions decrease CD36 expression and CD36 mediated-*Plasmodium* PEs phagocytosis through the downregulation of PPARγ

**Figure 1:**
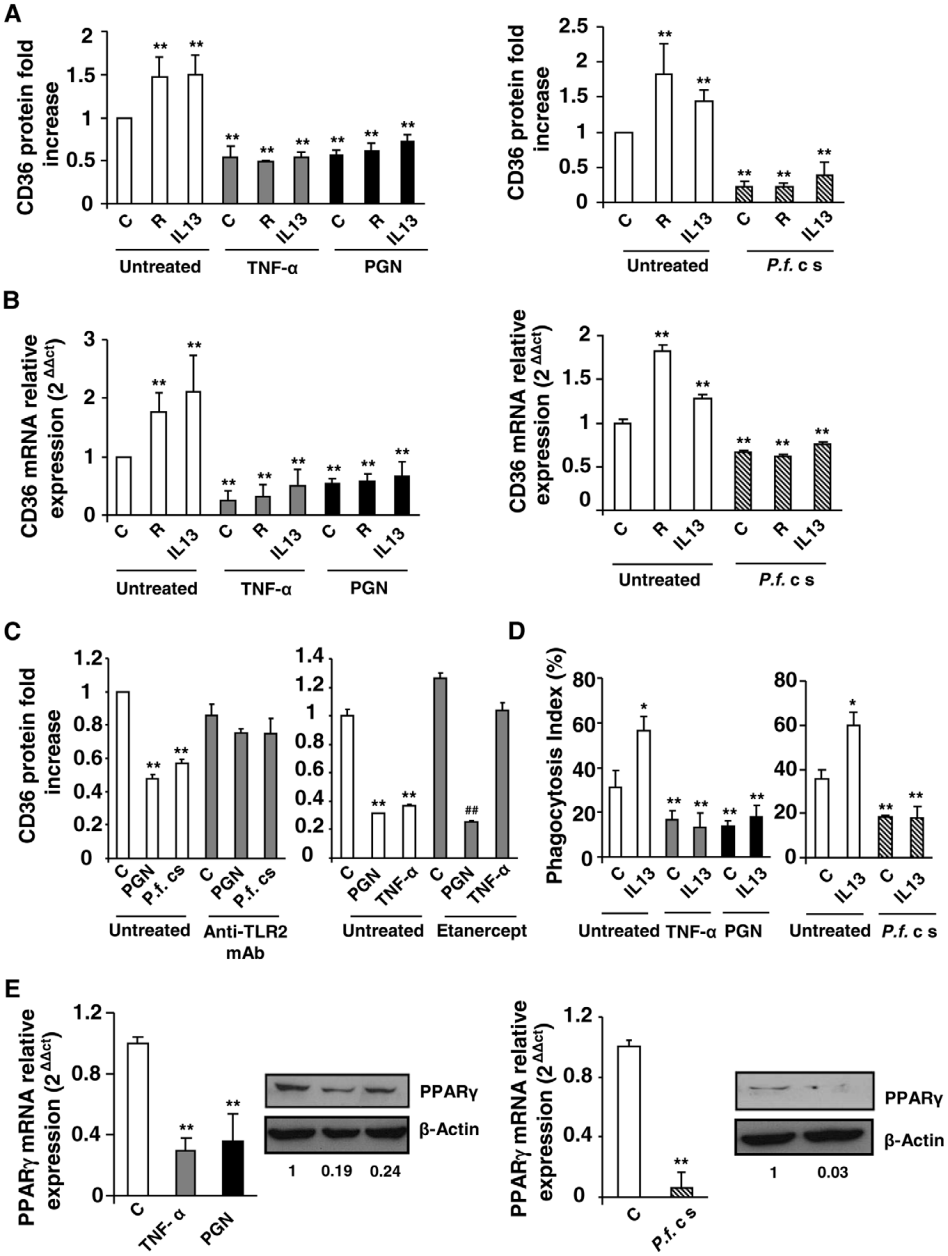
Inflammatory conditions decrease CD36 expression and CD36-mediated *Pf*PEs phagocytosis through downregulation of PPARγ. (A–B) CD36 protein and mRNA levels detected by flow cytometry or qRT-PCR on Swiss murine peritoneal macrophages firstly treated during 24 h with TNF-α (10 ng/mL), peptidoglycan (PGN) (1 µg/mL) or *Plasmodium falciparum* culture supernatant (*P.f.* c s) and incubated during 20 supplementary hours with rosiglitazone (R) (5 µM) or IL13 (50 ng/mL) for protein quantification or 5 supplementary hours for mRNA detection. Data are represented as a mean ± SD of three independent experiments. **p<0.01 compared with control cells (untreated). (C) CD36 protein level detected by flow cytometry on macrophages pre-incubated with a TL2 blocking monoclonal antibody (Anti-TLR2 mAb) (10 µg/mL) or Etanercept, a TNF-α inhibitor (10 µg/mL), and stimulated with TNF-α (10 ng/mL), PGN (1 µg/mL) or *P.f*. cs. Data are from a representative experiment performed in triplicate ± SD. **p<0.01 compared with control cells (untreated). ^##^p<0.01 compared with the respective control (Etanercept treated cells). (D) Phagocytosis index of *P. falciparum* unopsonized erythrocytes by Swiss murine macrophages stimulated as described in (A). Data are from a representative experiment performed in triplicate ± SD. The experiment was repeated three times. *p<0.05 and **p<0.01 compared with control cells (untreated). (E) PPARγ protein and mRNA levels in Swiss macrophages were determined by qRT-PCR after treatment of cells with TNF-α, PGN or *P.f.* cs. Data are represented as a mean ± SD of three independent experiments for mRNA quantification. **p<0.01 compared with control cells (C).

To determine the effect of inflammation on the modulation of CD36 expression, murine peritoneal macrophages were treated during 24 h for the quantification of CD36 protein level and during 5 h for mRNA level with TNF-α, peptidoglycan (PGN), or were incubated in presence of *P. falciparum* culture supernatant (*P.f.* cs) to mimic a more physiological malaria inflammatory context. The analysis of CD36 protein level on cells was evaluated on a selected R1 region, in which 92,2% of the cells were F4/80 and CD36 double-positive (Fig. S1A). Fig. 1A shows that TNF-α, PGN and *P.f.* cs treatments significantly decreased CD36 protein level. Consistent with this data, CD36 mRNA level was also downregulated following TNF-α, PGN and *P.f.* cs treatments (Fig. 1B). To assess whether TLR signaling directly leads to reduced CD36 expression or if the effects mediated by PGN or *P.f.* cs are dependent on TNF-α production, we evaluated CD36 protein level in presence of an anti-TLR2 antibody or etanercept, a potent TNF-α inhibitor. We demonstrated that PGN and *P.f*. cs treatments downregulate CD36 expression through TLR2 (Fig. 1C). The use of etanercept, unequivocally prove that the effects mediated by TLR2 activators on macrophage CD36 expression are independent of TNF-α production (Fig. 1C).

We then evaluated whether rosiglitazone and IL13, known to promote CD36 expression *via* an activation of the nuclear receptor PPARγ, could reverse the downregulation of CD36 receptor induced by inflammatory conditions. Murine peritoneal macrophages were firstly treated for 24 h with TNF-α, peptidoglycan (PGN) or *P.f.* cs and were then incubated for an additional time with rosiglitazone or IL13. Fig. 1A and FACS profiles (Fig. S1B, S1C) showed that rosiglitazone and IL13 increased CD36 protein level in noninflammatory conditions (control). However, in an inflammatory context both rosiglitazone and IL13 treatments failed to increase the CD36 protein and mRNA levels (Fig. 1A, 1B, S1B, S1C). These data demonstrate that inflammation negatively regulates CD36 expression and reveals the failure of PPARγ ligands to promote CD36 expression on macrophages in these conditions.

To determine whether these inflammatory conditions were also associated with an impaired *P. falciparum* clearance, the ability of macrophages to phagocytose *Pf*PEs was assessed. Fig. 1D showed a significantly reduced ability of macrophages to eliminate *Pf*PEs in inflammatory conditions (TNF-α, PGN, *P.f.* cs). In addition, IL13 was not able to restore the decrease of CD36-mediated phagocytosis of *Pf*PEs observed in inflammatory conditions (Fig. 1D). Therefore, the failure of PPARγ activators to promote CD36 expression and its *Pf*PEs phagocytosis-associated function in inflammatory conditions strongly suggests that PPARγ is no longer able to exert its transcriptional activity on the CD36 promoter.

To investigate whether the failure in CD36-dependent PPARγ transcriptional activity was correlated with a lower level of PPARγ expression, we evaluated PPARγ protein and mRNA levels in an inflammatory context. Interestingly, TNF-α, PGN and *P.f.* cs significantly decreased PPARγ protein and mRNA expressions (Fig. 1E). All these observations indicate that the inability of PPARγ ligands to enhance CD36 expression and its antimalarial associated functions in inflammatory conditions was associated with a marked decrease of PPARγ expression.

### Nrf2 activators promote CD36 expression and enhance CD36-mediated *Plasmodium falciparum-*PEs phagocytosis in inflammatory conditions

**Figure 2:**
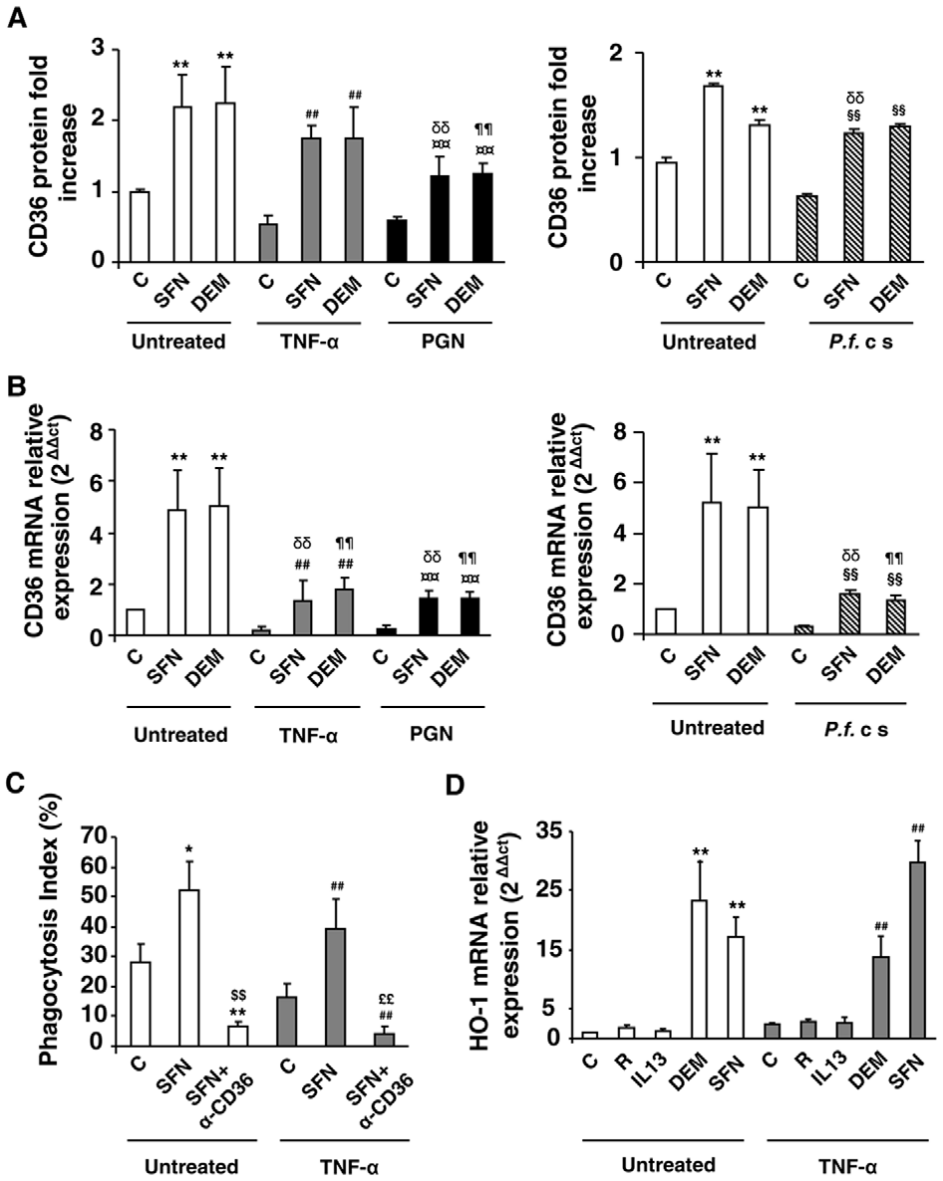
Nrf2 activators promote CD36 expression and enhance CD36-mediated *Pf*PEs phagocytosis in inflammatory conditions. (A–B) CD36 protein and mRNA levels detected by flow cytometry or qRT-PCR on Swiss murine peritoneal macrophages firstly treated during 24 h with TNF-α (10 ng/mL), PGN (1 µg/mL) or *P. falciparum* culture (*P.f.* c s) and then incubated during 20 h with sulforaphane (SFN) (10 µM) or diethylmaleate (DEM) (100 µM) for protein quantification or 5 supplementary hours for mRNA detection. Data are represented as a mean ± SD of three independent experiments. **p<0.01 compared with the respective control (untreated); ^##^p<0.01 compared with the respective control (TNF-α treated cells); ^¤¤^p<0.01 compared with the respective control (PGN treated cells); ^§§^p<0.01 compared with the respective control (*P.f*. cs treated cells); ^δδ^ p<0.01compared with control SFN-treated cells; ^¶¶^ p<0.01compared to control DEM-treated cells. (C) Phagocytosis index of *Pf* PEs by Swiss murine macrophages stimulated as described in (A). Data are represented as a mean ± SD of three independent experiments. *p<0.05 and **p<0.01 compared with the respective control (untreated); ^##^p<0.01 compared with the respective control (TNF-α treated cells); ^$$^p<0.01 compared with the respective control (SFN treated cells); ^££^p<0.01 compared with the respective control (TNF+SFN treated cells) (D) HO-1 mRNA level in Swiss peritoneal macrophages determined by qRT-PCR after treatment of cells during 24 h with TNF-α (10 ng/mL) and then during 5 h with rosiglitazone (R) (5 µM), IL13 (50 ng/mL) or SFN (10 µM). Data are represented as a mean ± SD of three independent experiments. **p<0.01 compared with the respective control (untreated); ^##^p<0.01 compared with the respective control (TNF-α treated cells).

We hypothesized that the Nrf2 transcription factor, recently known to control the CD36 expression, could substitute the deficiency of PPARγ in acute inflammatory conditions to enhance the expression of the CD36 receptor. Murine macrophages were firstly treated over 24 h with TNF-α, PGN or *P.f.* cs and then incubated for an additional time with Nrf2 activators sulforaphane (SFN) or diethylmaleate (DEM). Fig. 2A and FACS profiles (Fig. S2A, S2B) demonstrate that sulforaphane (SFN) and diethylmaleate (DEM) increase the CD36 protein level both in noninflammatory (control) and in an inflammatory context (TNF-α, PGN or *Pf*cs). Consistent with this data, the same profiles of CD36 mRNA were obtained (Fig. 2B). Then, the study of CD36 protein level after the administration of Nrf2 or PPARγ activators before the onset of inflammation demonstrate that both Nrf2 and PPARγ ligands prevent the downregulation of CD36 expression. (Fig. S2C).

In parallel, we showed that Nrf2 activators also up-regulate PPARγ mRNA levels in an Nrf2-dependent manner (Fig. S2E, S2F). However, no synergistic effect between PPARγ and Nrf2 activators has been observed *in vitro* on macrophage CD36 expression in inflammatory conditions (Fig. S2D).

We then explored whether this CD36 up-regulation by Nrf2 activators during inflammatory conditions could restore the decrease of CD36-mediated phagocytosis of *Pf*PEs observed during inflammation. Fig. 2C reveals that an SFN treatment both in noninflammatory (control) and inflammatory conditions (TNF-α) enhanced the phagocytosis of *Pf*PEs. These inductions were abolished by the use of a CD36 specific antibody (α-CD36), demonstrating that these phagocytic processes were dependent of the CD36 receptor.

To validate that the transcriptional activity of Nrf2 was still effective under inflammatory conditions, we next studied the modulation of HO-1 gene expression, a specific Nrf2 target gene [18]. Both in noninflammatory (control) and inflammatory (TNF-α) conditions, SFN and DEM significantly increased HO-1 mRNA expression, while PPARγ activators (IL13 or rosiglitazone) did not change HO-1 mRNA level (Fig. 2D), demonstrating that Nrf2 transcriptional activity was still efficient in inflammatory conditions. Altogether, these results suggest that the Nrf2 transcriptional factor could be involved in the regulation of CD36 expression and hence in CD36-mediated *Pf*PEs phagocytosis in inflammatory conditions.

### Nrf2 activators promote CD36 expression independently of PPARγ

**Figure 3:**
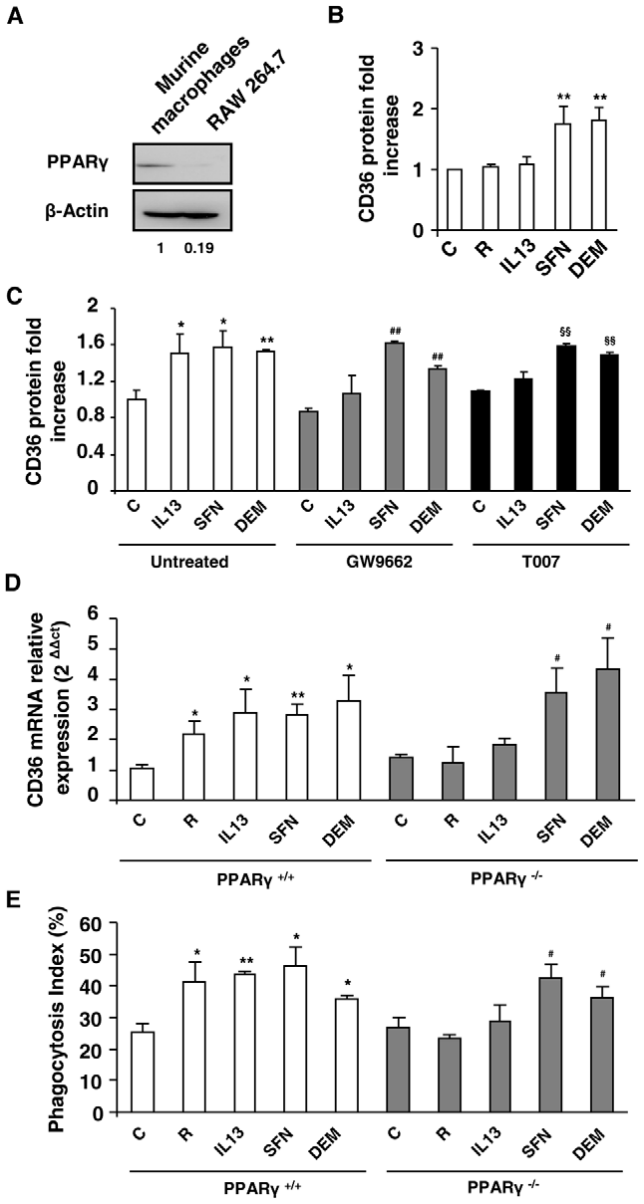
Nrf2 activators promote CD36 expression independently of PPARγ. (A) PPARγ protein level on Swiss murine peritoneal macrophages and on RAW264.7 cells. The experiments were repeated three times. (B) CD36 protein level on murine RAW264.7 cells after treatment of cells with IL13 (50 ng/mL), rosiglitazone (R) (5 µM), sulforaphane (SFN) (10 µM) or diethylmaleate (DEM) (100 µM). Data are represented as a mean ± SD of three independent experiments. **p<0.01 compared with control cells. (C) CD36 protein level detected by flow cytometry on Swiss murine peritoneal macrophages firstly treated during 1 h with the PPARγ antagonists GW9662 (5 µM) and T007 (2 µM) and then incubated during 20 h with IL13, SFN or DEM. Data are represented as a mean ± SD of three independent experiments. **p<0.01 compared with the respective control (untreated); ^##^p<0.01 compared with the respective control (GW9662 treated cells); ^§§^p<0.01 compared with the respective control (T007 treated cells). (D) CD36 mRNA level on PPARγ^+/+^ and PPARγ^−/−^ C57BL/6 murine peritoneal macrophages after treatment of cells with rosiglitazone, IL13, SFN or DEM. Data are from a representative experiment performed in triplicate ± SD. The experiment was repeated three times. *p<0.05 and **p<0.01 compared with the respective control (PPARγ^+/+^); ^##^p<0.01 compared with the respective control (PPARγ^−/−^). (E) Phagocytosis index of *P. falciparum* unopsonized erythrocytes by murine PPARγ^+/+^ and PPARγ^−/−^ C57BL/6 macrophages stimulated as described in (E). Data are from a representative experiment performed in triplicate ± SD. The experiment was repeated three times. *p<0.05 and **p<0.01 compared with the respective control (PPARγ^+/+^); ^#^p<0.05 compared with the respective control (PPARγ^−/−^).

To establish that the Nrf2 transcription factor may promote CD36 expression in absence of the PPARγ nuclear receptor, we performed experiments on the RAW 264.7 macrophage murine cell line which expresses a very low level of PPARγ, as demonstrated in Fig. 3A. Fig. 3B shows that PPARγ specific activators, IL13 and rosiglitazone, did not change the CD36 protein level, whereas the Nrf2 activators (SFN or DEM) strongly enhanced CD36 protein expression (Fig. 3B). These results show that in absence of PPARγ, Nrf2 activators are able to promote CD36 expression.

To further confirm that PPARγ was not involved in the regulation of CD36 by Nrf2 activators, murine macrophages were incubated in the presence of GW9662 or T007, two specific irreversible antagonists of PPARγ. Fig. 3C shows that the macrophages treated by GW9662 or T007 failed to up-regulate CD36 expression after exposure to IL13. In contrast, GW9662 or T007 treatments did not affect CD36 over-expression observed with SFN or DEM treatments. Altogether these data prove that the induction of CD36 by Nrf2 activators is independent of PPARγ.

Finally, to unequivocally prove that Nrf2 activators could promote CD36 expression in absence of PPARγ, we studied CD36 mRNA expression in macrophages in which PPARγ had been selectively disrupted. As expected, the expression of CD36 was promoted by rosiglitazone, IL13, SFN or DEM treatments in macrophages from PPARγ^+/+^ mice. Interestingly, the CD36 over-expression following rosiglitazone or IL13 treatments failed in PPARγ deficient macrophages (PPARγ^−/−^), while SFN or DEM treatments enhanced CD36 expression in PPARγ^−/−^ cells (Fig. 3D). In line, the *Pf*PEs phagocytosis level by PPARγ^−/−^ macrophages was only enhanced following SFN or DEM treatments and not by rosiglitazone or IL13 (Fig. 3E). Altogether these data establish that only Nrf2 activators contribute to the enhancement of CD36 expression and CD36 mediated-*P. falciparum* phagocytosis in absence of PPARγ.

### Nrf2 activation is involved in CD36 induction during inflammatory conditions

**Figure 4:**
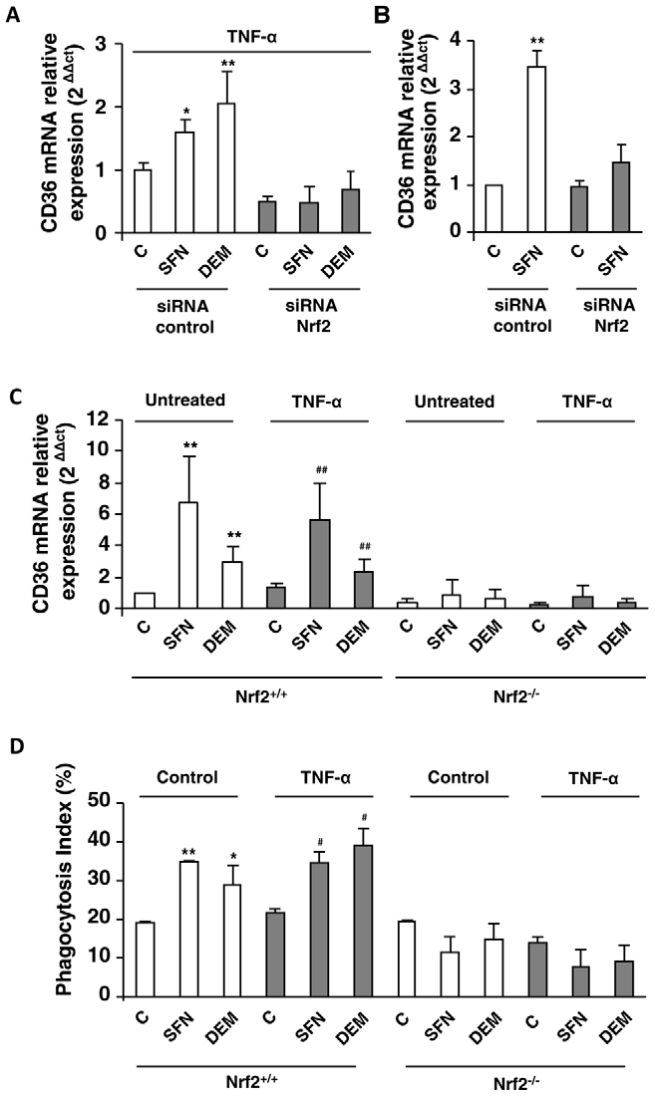
Nrf2 transcription factor is involved in CD36 overexpression during inflammatory processes. CD36 mRNA level on Swiss peritoneal macrophages treated during 24 h with TNF-α (10 ng/mL) (A) and on RAW264.7 cells (B) and transfected with siRNA targeting Nrf2 (siRNA Nrf2) or control siRNA (siRNA control) and stimulated with sulforaphane SFN (10 µM) or diethylmaleate (DEM) (100 µM). Data are represented as a mean ± SD of three independent experiments. *p<0.05 and **p<0.01 compared with the respective control (cells transfected with siRNA control), (C) CD36 mRNA level on Nrf2^+/+^ and Nrf2^−/−^ C57BL/6 murine peritoneal macrophages after treatment during 24 h with TNF-α and then incubated during 5 h with SFN or DEM. Data are from a representative experiment performed in triplicate ± SD. The experiment was repeated three times **p<0.01 compared with the respective control (Nrf2^+/+^), ^##^p<0.01 compared with the respective control (Nrf2^+/+^ cells treated with TNF-α). (D) Phagocytosis index of *Pf*PEs by murine Nrf2^+/+^ and Nrf2^−/−^ C57BL/6 macrophages stimulated as described in (C). Data are from a representative experiment performed in triplicate ± SD. The experiment was repeated three times. *p<0.05 and **p<0.01 compared with the respective control (Nrf2^+/+^ control cells); ^#^p<0.05 compared with the respective control (Nrf2^+/+^ cells treated with TNF-α).

To confirm the specific involvement of Nrf2 transcription factor in the regulation of CD36 expression under inflammatory conditions, TNF-α activated macrophages were transiently transfected with siRNA specifically targeting Nrf2. As predicted, the siRNA-mediated knock down of Nrf2 decreased HO-1 expression in TNF-α treated macrophages following SFN or DEM treatments (Fig. S3A, S3B). Interestingly, the increase of CD36 mRNA level both in TNF-α treated macrophages (Fig. 4A) and RAW cells (Fig. 4B) following SFN or DEM treatments was abolished after transfection with siRNA targeting Nrf2. These data strongly suggest that the up-regulation of CD36 in inflammatory context is mediated by Nrf2 transcription factor.

To consolidate our hypothesis, we studied CD36 and HO-1 mRNA levels in macrophages from Nrf2^−/−^ mice. The induction of CD36 (Fig. 4C) and HO-1 (Fig. S3C) mRNA levels by SFN or DEM treatments in macrophages from Nrf2^+/+^ mice both in normal (control) and in inflammatory conditions (TNF-α) was not observed in macrophages from Nrf2^−/−^ mice. Consistently, the levels of *P. falciparum* phagocytosis were not enhanced by SFN or DEM treatments in Nrf2^−/−^ macrophages (Fig 4D). Altogether, these data clearly revealed that Nrf2 plays a crucial role in the activation of CD36 macrophage gene expression and in its phagocytosis-associated functions during acute inflammatory processes.

**Figure 5:**
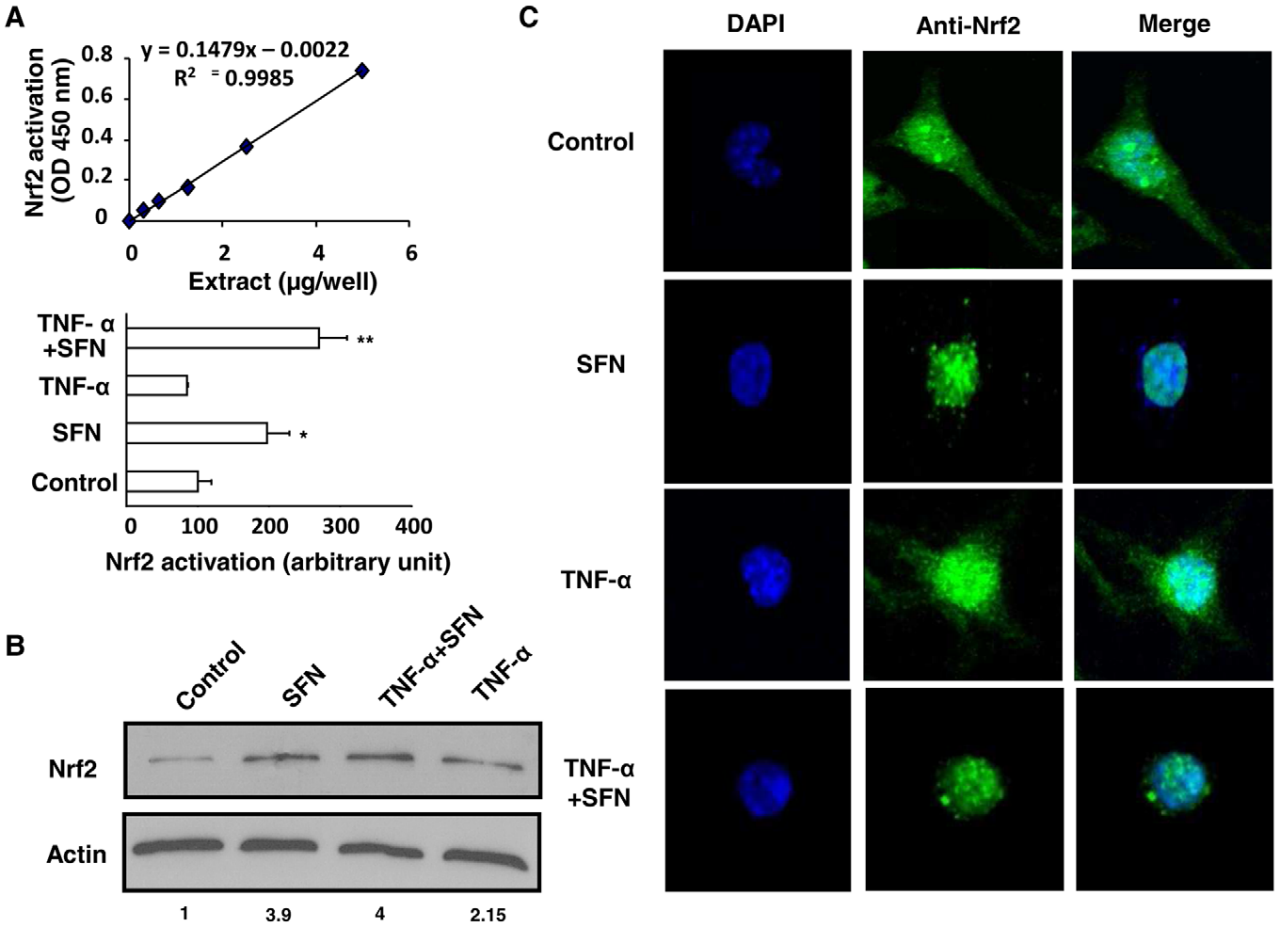
Nrf2 activation by sulforaphane occurs even under inflammatory conditions. (A) Quantification of the ARE-nuclear binding of Nrf2 by DNA-binding ELISA TransAM kit after treatment of Swiss peritoneal macrophages with TNF-α during 24 h and then during 1 h with SFN. The data are expressed as relative arbitrary units. Data are from a representative experiment performed in triplicate ± SD. The experiment was repeated three times. *p<0.05 and **p<0.01 compared with control cells. (B) Nrf2 protein level in nuclei extracts of cells described in (A) (C) Confocal laser microscopy on Swiss macrophages described in (A). The blue color represents the nucleus; the green color represents Nrf2. Merged images of the blue and green colors are shown in the right-hand panels. The data are representative of three independent experiments.

To evaluate the DNA-binding activity of Nrf2 transcription factor in inflammatory conditions, a DNA-binding ELISA-based assay using a specific Nrf2 antibody was performed. Fig. 5A demonstrates that Nrf2 was specifically activated by SFN treatment and bound to its ARE-binding sequence both in noninflammatory and in inflammatory (TNF-α) conditions. Fig. 5B reveals that the Nrf2 protein level was increased in the nucleus of SFN-treated cells both in noninflammatory and in inflammatory (TNF-α) conditions. Then, to evaluate this nuclear localization of Nrf2 following SFN treatment, confocal laser scanning microscopy analysis was performed. Nrf2 transcription factor was localized both in the nucleus and in the cytoplasm of control and TNF-α treated cells (Fig. 5C). Interestingly, Nrf2 was exclusively located in the nucleus following SFN treatment both in noninflammatory and in inflammatory (TNF-α) conditions. Altogether these results confirm that after SFN treatment Nrf2 translocates to the nucleus and exerts its transcriptional activity during inflammatory conditions.

### Nrf2 activators up-regulate CD36 and increase phagocytosis of *P. falciparum*-PEs on human monocytes-derived macrophages in inflammatory conditions

**Figure 6:**
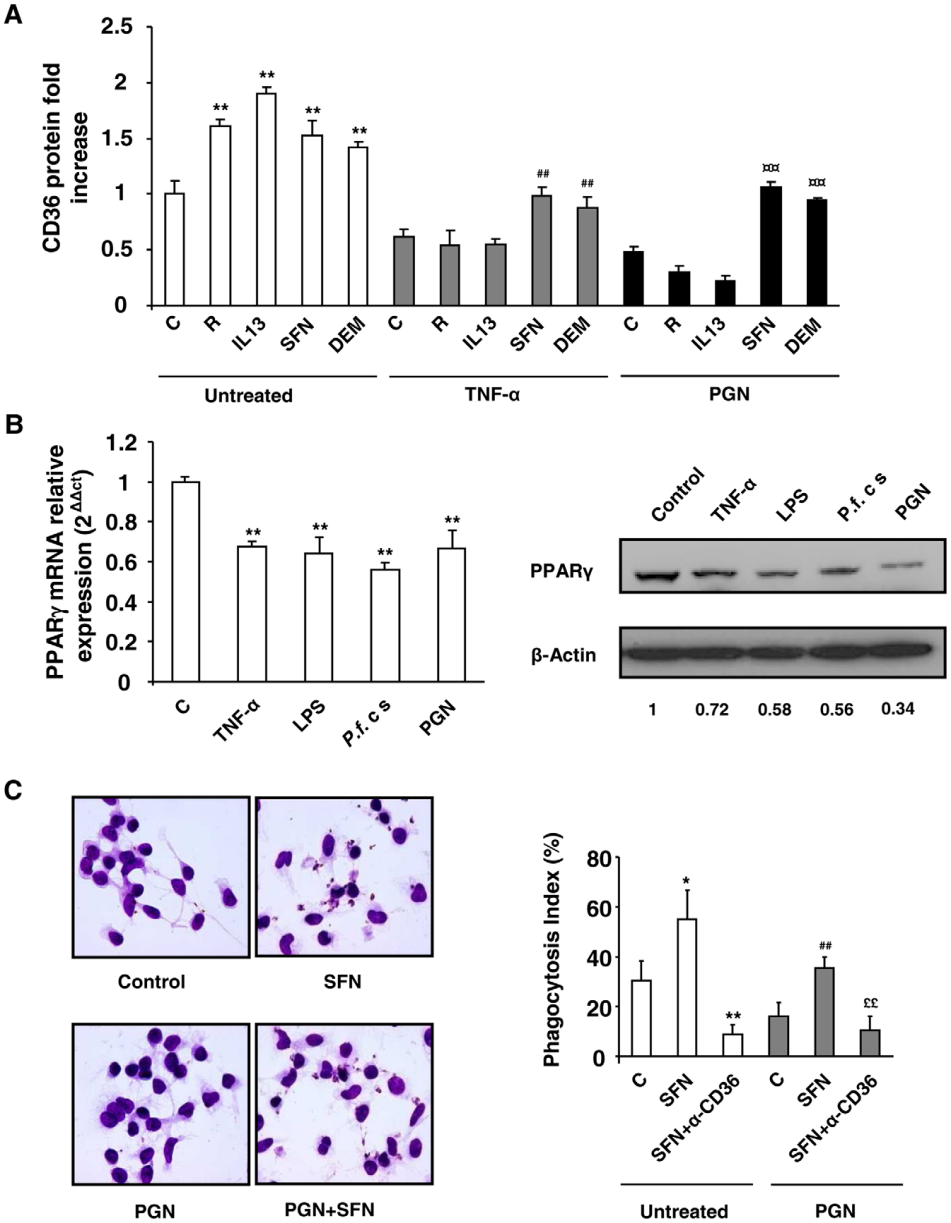
Nrf2 activators promote CD36 and increase *P.falciparum* clearance by human MDMs in inflammatory conditions. (A) CD36 protein level detected by flow cytometry on human-monocytes derived macrophages (hMDMs) firstly treated during 24 h with TNF-α (10 ng/mL) or PGN (1 µg/mL) and then incubated during 20 h with rosiglitazone (R) (5 µM), IL13 (50 ng/mL), SFN (10 µM) or DEM (100 µM). Data are represented as a mean ± SD of three independent experiments. **p<0.01 compared with the respective control (untreated); ^#^p<0.05 compared with the respective control (TNF-α treated cells); ^¤¤^p<0.01 compared with the respective control (PGN treated cells). (B) PPARγ protein and mRNA levels on hMDMs treated with TNF-α, LPS, *P.f*. cs and PGN. PPARγ western blot is representative of three independent experiments and qPCR data are represented as a mean ± SD of three independent experiments. **p<0.01 compared with the respective control (untreated). (C) Phagocytosis index of *P. falciparum* unopsonized erythrocytes by hMDMs stimulated as described in (A). Data are represented as a mean ± SD of three independent experiments. **p<0.01 compared with the respective control (untreated); ^##^p<0.01 compared with the respective control (PGN treated cells); ^$$^p<0.01 compared with the respective control (SFN treated cells); ^££^p<0.01 compared with the respective control (TNF+SFN treated cells).

To extend our results to human monocytes-derived macrophages (hMDMs), we evaluated the CD36 protein level on inflammatory hMDMs following rosiglitazone, IL13, SFN or DEM treatments. Cells were gated on the R1 region, corresponding to the hMDMs population highly expressing CD36 (Fig. S4C). TNF-α, PGN and *P.f.* cs downregulated CD36 protein and mRNA levels on hMDMs (Fig. 6A, S4A, S4B). The increase of CD36 protein level in control hMDMs following treatments by rosiglitazone, IL13, SFN and DEM was only observed in TNF-α and PGN treated hMDMs after SFN or DEM treatments. Indeed, rosiglitazone and IL13 did not promote CD36 protein level in these hMDMs (Fig. 6A, S4D). These data confirm in humans that during inflammatory processes only Nrf2 activators were able to promote CD36 expression. The failure to promote CD36 expression on hMDMS *via* the PPARγ signaling pathway was associated with a marked reduction of PPARγ mRNA and protein levels (Fig. 6B) during acute inflammatory processes.

Finally, the ability of hMDMs to mediate the phagocytosis of *Pf*PEs under acute inflammatory processes was determined. Fig. 6C reveals that SFN treatment promoted the phagocytosis of *P. falciparum* both in noninflammatory (control) and inflammatory (PGN) hMDMs. Phagocytosis both in normal and inflammatory contexts was significantly inhibited by a CD36 specific antibody, demonstrating that the induction of *P. falciparum* phagocytosis by SFN treated hMDMs was dependent of CD36 receptor (Fig. 6C). These data indicate that in humans during inflammatory processes, only Nrf2 activators up-regulate CD36 expression on hMDMs and *P. falciparum* phagocytosis-associated function.

### Nrf2 but not PPARγ activators improve *in vivo* severe malaria outcome

Since we have demonstrated that Nrf2 regulates similarly CD36 receptor expression in inflammatory conditions both in human monocytes-derived macrophages and in Swiss murine peritoneal macrophages, we developed an induced-inflammatory severe malaria model in Swiss genetic background to validate that Nrf2 could be a therapeutic target in the prevention of severe malaria.

**Figure 7:**
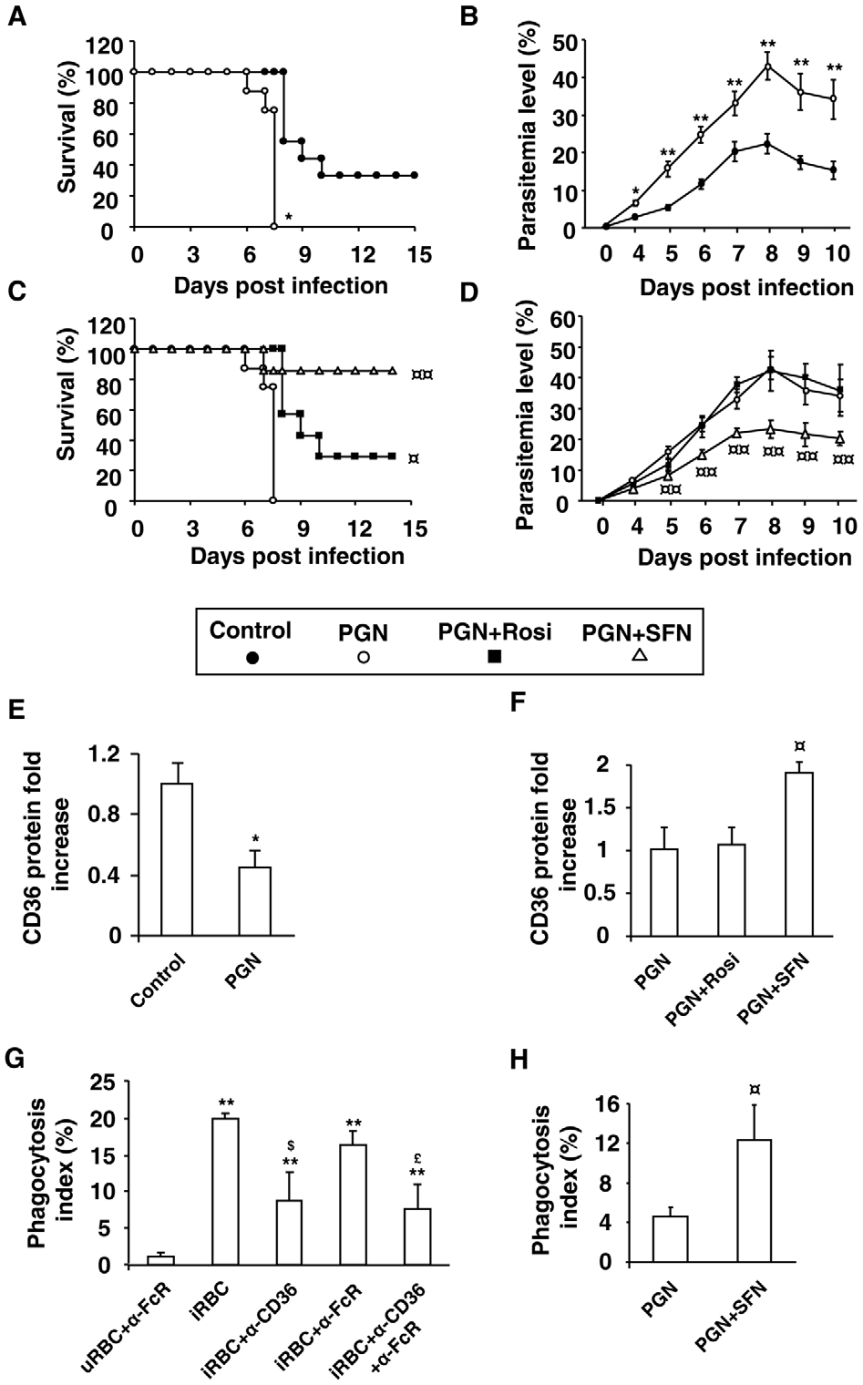
Nrf2 activator sulforaphane improves the outcome of induced-severe malaria trough a reduction in parasite burden. Swiss mice receiving PGN (200 µg/mouse) (subcutaneous route) two days before infection and d,L-sulforaphane (75 mg/kg) or rosiglitazone (3 mg/kg) (oral route) during five days postinfection were infected with 1×10^6^ *Plasmodium berghei* parasites *via* intra-peritoneal injection. (A) and (C) Survival was assessed twice daily. **p<0.01 compared with untreated mice (control), ^¤¤^p<0.01 compared with PGN-treated mice. (B) and (D) Parasitemia levels were assessed daily. **p<0.01 compared with untreated macrophages (control); ^¤^p<0.05 and ^¤¤^p<0.01 compared with PGN-treated cells. (E) and (F) CD36 expression on macrophages was measured the day of infection by flow cytometry on three independent mice. *p<0.05 compared with untreated macrophages (control); ^¤^p<0.05 compared with PGN-treated cells. (G) Phagocytosis index of *P. berghei* unopsonized erythrocytes by Swiss murine macrophages incubated with FcR blocking antibodies (20 µg/mL) and CD36 blocking antibodies (10 µg/mL). Data are presented as mean ± SD of one experiment performed in triplicate. **p<0.01 compared to uRBC+α−FcR, ^$^p<0.05 compared with iRBC+α−CD36, ^£^p<0.05 compared with iRBC+α−FcR. (H) Phagocytosis index of *P. berghei* unopsonized erythrocytes by 3 days-infected macrophages from Swiss mice treated with PGN and SFN as described above. ^¤^p<0.05 compared with PGN-treated macrophages.

To establish this malaria model, Swiss mice were pre-treated with peptidoglycan (PGN), a TLR2 activator, and then infected with *P. berghei*. As expected, PGN-treated mice had a significantly lower survival rate compared with control mice (p = 0,043) which did not succumb in the early phase of infection, demonstrating that PGN-induced inflammatory processes strongly worsen the outcome of infection in the Swiss mice model (Fig. 7A). In addition, PGN treated mice presented significant higher parasitemia levels than control animals (Fig. 7B), but did not succumb from anemia (data not shown). Altogether, these results indicate that installed acute inflammatory processes before infection by *P. berghei* in mice clearly worsen the severity of the infection.

To determine whether the PGN-induced increase in parasite burden observed in our severe malaria model was associated with macrophage CD36 downregulation, we evaluated the CD36 protein level on peritoneal macrophages. PGN treatment significantly decreased CD36 protein level (Fig. 7E). Altogether these data demonstrate that inflammatory processes induced by PGN treatment in infected mice downregulate the macrophage CD36 expression and contribute to worsen the severity of malaria infection in mice.

To assess whether Nrf2 activators or PPARγ ligands were able to improve the outcome of severe malaria, PGN-induced severe malaria mice were treated with SFN or rosiglitazone. The *in vivo* oral treatment of mice with SFN initiated the day of infection and followed 5 days post infection greatly increases the survival rates of mice (p = 0,0001) (Fig. 7C) and contributes to limit the parasite burden in the first days of infection (Fig. 7D). The rosiglitazone treatment increases the survival rates of PGN treated mice (p = 0,032) (Fig. 7C) and does not affect their blood parasitemia level (Fig. 7D). Interestingly, the CD36 protein level on macrophages only increased after *in vivo* SFN treatment (Fig. 7F), demonstrating that in an *in vivo* acute inflammatory context only Nrf2 activators and not PPARγ ligands are able to up-regulate CD36 expression. Finally, we demonstrate that macrophages from infected SFN-treated mice enhance *P. berghei* clearance (Fig. 7H). The use of anti-CD36 and anti-FcR antibodies demonstrates that *P. berghei*-PEs were internalized in a CD36 dependent manner (Fig. 7G). Altogether, these results strongly suggest that targeting Nrf2 *in vivo* contributes to improve the outcome against inflammatory-induced severe malaria in mice.

## Discussion

Mononuclear phagocytes play an important role in the clearance of blood-stage malaria parasites resulting in an early control of parasite proliferation during acute infection [5], [20]. The importance of CD36 receptor expression on macrophages in malaria parasite clearance and in the regulation of parasite-induced inflammatory processes has been demonstrated both *in vitro* and *in vivo* on CD36^−/−^ mice [5]. Recent data provide evidence that rosiglitazone PPARγ ligand can *in vitro* and *in vivo* improves the outcome of experimental malaria in mice, enhancing CD36-mediated PEs phagocytic processes and limiting parasite-induced inflammatory processes [9]. Nevertheless, much of the pathology associated with malaria infections is a result of excessive and uncontrolled production of proinflammatory markers [11], [21]. In this study, we demonstrated that inflammatory processes induced by TNF-α or TLR2 ligands downregulate CD36 expression on Swiss murine and human macrophages. These data are consistent with other studies, highlighting the deleterious effect of LPS, IFNγ or TNF-α treatment on CD36 expression [13], [14], [22], [23]. We also show that a similar CD36 downregulation was observed on murine and human macrophages following *Pf*cs treatment, which contains soluble factors released from infected erythrocytes rupture such as *Pf*GPI anchors (glycophosphatidylinositol), described as TLR2 ligands [24]. In addition, we have previously demonstrated that circulating human monocytes presented a loss of CD36 expression during the acute phase of plasmodial infection [12]. In parallel, we demonstrated that PGN and *P.f*. cs treatments downregulate CD36 expression specifically through TLR2. Surprisingly, the TLR2 activators-mediated CD36 dowregulation is independent of TNF-α release. In addition, we demonstrate in this study that this decrease in CD36 expression on macrophages impairs CD36-mediated *Pf*PEs phagocytosis. Consistent with these data, prostaglandin E2, a pro-inflammatory eicosanoid, was shown to downregulate CD36 expression on macrophage directly resulting in a reduced CD36-phagocytic ability leading to the development of endometriosis [25].

PPARγ ligands thiazolidinediones and IL4 or IL13, two Th2 cytokines known to activate PPARγ, were previously shown *in vitro* to promote CD36 expression and enhance CD36-mediated PEs phagocytosis [4], [6], [8], [9], [26]. Surprisingly, our current results revealed that PPARγ ligands and IL13 have no effect on the modulation of CD36 expression in inflammatory macrophages. Consistent with this, TNF-α was previously shown to downregulate CD36 expression on human monocytes involving a direct reduction in PPARγ activation [13]. In line with these results, it has been reported that inflammatory processes reduce PPARγ expression and activation, highlighting the deleterious effect of inflammatory processes on PPARγ [27]–[29]. Collectively, our data provides compelling evidence that inflammatory processes negatively regulate the expression of PPARγ in swiss murine macrophages and human monocyte-derived macrophages, resulting in a failure to trigger this pathway to promote CD36 expression and *Plasmodium* clearance. Interestingly, inflammatory stimuli did not negatively regulate the expression of PPARγ and hence CD36 expression in C57BL/6 murine macrophages, suggesting the importance of genetic background in their regulation. Therefore, it seems that the Swiss mice model better mimics the human model. Indeed, inflammatory processes inhibit PPARγ and CD36 in hMDMS, and monocytes from *Plasmodium*-infected patients exhibit a CD36 dowregulation [12].

In our study, we demonstrated that PPARγ^−/−^ macrophages did not present a totally abolished CD36 phenotype, suggesting the existence of alternative pathways controlling CD36 expression on macrophages. We also previously demonstrated that the increase of the CD36 receptor following an anti-TNF-α antibody treatment occurred independently of PPARγ and involved radical oxygen species production (ROS) *via* NADPH oxidase activation [13]. The ROS are potent inducers of Nrf2 activation, a transcription factor involved in the prevention of severe inflammatory diseases [16]. Interestingly, this transcription factor was shown to play an important role in the regulation of CD36 expression on murine macrophages [18]. Thus, we postulated that Nrf2 transcription factor might substitute PPARγ to promote CD36 expression during acute inflammatory processes. Consistent with this hypothesis, we demonstrated that SFN or DEM, Nrf2 activators, upregulate CD36 expression both on murine and hMDMs during inflammatory processes. This CD36 overexpression leads to a higher elimination rate of *Pf*PEs by macrophages. CD36 induction following SFN or DEM treatments was shown to be PPARγ independent and Nrf2 dependent. Altogether, these data unequivocally demonstrate that the Nrf2 transcription factor regulate CD36 expression in absence or in the presence of PPARγ nuclear receptor.

Serghides *et al.* have recently focused on the effects of PPARγ ligand treatment in an inflammatory cerebral malaria murine model. The rosiglitazone treatment improves the survival rate of mice with cerebral malaria. Nevertheless, time course administration of rosiglitazone revealed that the rosiglitazone treatment was a lot more efficient when administered one week before infection than one day after the onset of the disease [9]. In our induced-inflammatory severe malaria model, we demonstrated that rosiglitazone administered after the onset of infection, when the inflammatory processes are already triggered, has a very slight effect compared with SFN effect on mice survival rate and no effect on the modulation of parasitemia level. The positive *in vivo* effect of rosiglitazone on Swiss mice survival may be associated to a refractory population of macrophages to the inflammatory mediated CD36 downregulation. The differences observed between our study and the data published by the Kain's group are certainly related to the genetic background of the murine models used. Indeed, we did not in fact observe *in vitro* the downregulations of PPARγ and CD36 on macrophages from C57BL/6 mice in inflammatory conditions while PPARγ and CD36 were greatly impaired in swiss macrophages, as in hMDMs. These data suggest that the benefit of using rosiglitazone depends on the modulation of PPARγ expression which seems to be dependent on individual genetic background. The variability of rosiglitazone effectiveness in inflammatory processes has already been observed in other studies. Preventive administration of thiazolidinediones did in fact provide beneficial effects in murine models of ulcerative colitis but was less efficient when administered after the onset of the disease because PPARγ was shown to be downregulated by colitis-induced inflammatory processes [30]. Similar results were observed in human patients with colitis, which present a modest improvement in the outcome of the disease after rosiglitazone treatment [31]. This rosiglitazone inefficiency could be directly related to an impairment of PPARγ expression observed in patients with inflammatory colitis [32].

Interestingly, as opposed to the rosiglitazone treatment which slightly decreases severe malaria infection, we demonstrated here for the first time that the SFN, an Nrf2 activator, strongly contributes to control the elevation of parasite burden and hence consistently improves the outcome of severe inflammatory induced-malaria. This was correlated to an induction of CD36 expression on macrophage following SFN treatment and with a higher phagocytic capacity for PEs. In addition, SFN treatment did not alter major pro- and anti-inflammatory cytokines involved in malaria physiopathology (Fig. S5A, S5B), suggesting that SFN-induced protection is essentially due to the uptake of parasites through CD36 receptor. In addition, we showed that SFN treatment also reduces parasitemia in Swiss infected mice without prior PGN treatment (data not shown), indicating that the Nrf2 pathway is also important under more natural infection conditions.

Recently, a clinical study in humans described the effectiveness of rosiglitazone as an adjunct treatment to standard therapy for non severe malaria [33]. Nevertheless, although the effects of rosiglitazone in combination with conventional antimalarial treatments were effective in a non severe *P. falciparum* infection associated with moderated and controlled inflammatory processes, one question may subsist on the effectiveness of rosiglitazone when administered during severe malaria when acute inflammatory processes are engaged. We showed on human inflammatory MDMs that the rosiglitazone treatment was ineffective on CD36-mediated parasite clearance due to a downregulation of PPARγ. Interestingly, the Nrf2 activators treatments were able to promote CD36 expression on human MDMs and hence participate actively to the clearance of *Plasmodium*.

In conclusion, the present results provide direct evidence that inflammatory processes and particularly malaria parasite-induced inflammatory processes impair CD36 expression on Swiss murine as on human MDMs and CD36-mediated phagocytosis, favoring the worsening of malaria infection. This observation is correlated to a failure in PPARγ expression and activation. However, in inflammatory conditions, we demonstrated that Nrf2 pathway controls CD36 expression and improves the outcome of severe malaria independently of PPARγ. Thus, the results suggest the possibility that Nrf2 may be a therapeutic target for the control of severe malaria.

## Materials and Methods

### Ethics statement

This study was carried out in accordance with Approval No B3155503 and all animal experiments followed the guiding principles of animal care and use defined by the Conseil Scientifique du Centre de Formation et de Recherche Experimental Médico Chirurgical (CFREMC) with the rules of Decree 87–848 dated 10/19/1987 (modified by Decree 2001-464 and Decree 2001-131 relative to European Convention, EEC Directive 86/609 dated 24/11/1986). The experiments were approved by the ethics board of the Midi-Pyrénées ethic committee for animal experimentation (Experimentation permit number 31-067).

Monocytes were obtained from healthy blood donors (Etablissement Français du Sang, Toulouse). Written informed consents were obtained from the donors under EFS contract n°21/PVNT/TOU/UPS04/2010–0025. Following articles L1243-4 and R1243-61 of the French Public Health Code, the contract was approved by the French Ministry of Science and Technology (agreement n°AC 2009-921).

### Animals

Female Swiss and C57BL/6 transgenic 6–12-week-old mice were used both for *in vitro* and *in vivo* experiments. The generation of the PPARγ^−/−^ [8] and Nrf2^−/−^ [34] mouse lines were previously described.

### Mouse peritoneal macrophages isolation

Resident peritoneal cells were harvested by washing the peritoneal cavity with sterile NaCl 0,9%. Collected cells were centrifuged and the cell pellet was suspended in Macrophage-Serum Free Medium (M-SFM) (Gibco Invitrogen). Cells were allowed to adhere for 2 h at 37°C, 5% CO_2_. Non-adherent cells were then removed by washing with PBS.

### Human monocytes isolation and differentiation into macrophages

Human peripheral blood mononuclear cells were isolated from the blood of healthy volunteers by a density gradient centrifugation method on Lymphoprep (Abcys). Monocytes were isolated by adherence to plastic for 2 h in M-SFM at 37°C, 5% CO_2_. Monocytes were cultured for 5–7 supplementary days in M-SFM containing 50 ng/mL M-CSF (eBiosciences) to allow for differentiation into human monocyte-derived macrophages.

### *P. falciparum* culture and supernatant collection

The laboratory *P. falciparum* strain FcB1-Columbia presenting the phenotype Knobs+ at the erythrocyte surface was continuously cultured according to Trager and Jensen [35] with modifications [36]. To obtain *P. falciparum* culture supernatant (*P.f.* cs) for *in vitro* stimulations, parasites were highly synchronized by 5% D-Sorbitol treatment (Sigma). After schizont stage-infected erythrocyte rupture occurred, the culture medium was collected and centrifuged before being used for *in vitro* experiments. For phagocytosis assays, trophozoite-stage infected erythrocytes were washed and used at a PEs macrophage ratio of 20∶1. For *P.berghei* phagocytosis experiments, we used a PEs macrophage ratio of 10∶1.

### Reagents

Murine peritoneal macrophages and hMDMs were stimulated by TNF-α (10 ng/mL) (eBiosciences), LPS (100 ng/mL) (Sigma Aldrich), Peptidoglycan (PGN) (1 µg/mL) (Sigma Aldrich), rosifoglitazone (5 µM) (Cayman Chemical), IL-13 (50 ng/mL) (eBiosciences), sulforaphane (SFN) (10 µM) (Sigma Aldrich), diethylmaleate (DEM) (100 µM) (Sigma Aldrich), and by 500 µL of *P.f.* cs. Macrophages were incubated with the specific inhibitors of PPARγ, GW9662 (5 µM) and T0070907 (2 µM) (Cayman Chemical), 30 min before the addition of PPARγ or Nrf2 activators.

### Flow cytometry assay

Murine macrophage surface expression of CD36 was detected as previously described [37]. Briefly, murine macrophage CD36 expression was detected using a PE-monoclonal CD36 antibody (Santacruz, sc-13572) and compared with an irrelevant appropriate isotype control (Santa Cruz, sc-3600). hMDMs macrophages were stained with mouse IgM,κ CD36-APC antibody (BD Pharmingen). A mouse IgM,κ isotype-matched antibody conjugated to APC (BD Pharmingen) was used as a control. A minimum population of 3000 cells was analyzed for each data point. All analyses were performed on a FACScan using CellQuestPro software (Becton Dickinson).

### Quantitative real-time PCR experiments

RNA and cDNA preparation were as described [38]. Quantitative RT-PCR was performed on a LightCycler 480 system using LightCycler 480 SYBR GREEN I MASTER (Roche Diagnostics). β-actin was used as the invariant control. The sequences of primers were listed in supplementary Table S1. The N-fold differential expression of mRNA gene samples was expressed as 2^ΔΔCt^.

### Fluorescence imaging confocal microscopy

Cells were fixed with PBS containing 4% paraformaldehyde and were then incubated in a glycine solution (100 mM). After permeabilization and blocking, cells were then incubated overnight at 4°C with anti-Nrf2 antibody (Santacruz, sc-13032). Cells were then incubated with Alexa 488-conjugated anti rabbit antibody (Invitrogen) for 1 h at room temperature. Nuclei were stained with DAPI. Treated cells were covered with glass slips using Perma Fluor (Thermo Scientific). All microscopy imagery was performed with a LEICA SP2 laser scanning confocal microscope. For each condition, 40–50 cells were analyzed. The staining is representative of three independent experiments.

### Phagocytosis assays

Phagocytosis assays were performed as already described [8]. The phagocytic index was calculated as the percentage of macrophages with PEs phagocytosed.

### Transfection assays

Nrf2 siRNA (Santacruz sc-37049) and control siRNA (Santacruz sc-37007) were transfected into RAW cells or Swiss murine peritoneal macrophages using Lipofectamine 2000 reagent (Invitrogen) as described in the manufacturer's protocol.

### Nuclear protein extraction and DNA-binding activity

Nuclear proteins were isolated with NE-PER kit (Thermo Scientific). Nrf2 TransAM ELISA-kit (Active Motif) was used to evaluate Nrf2 DNA-binding activity. The final A_450_ was read on a microplate reader (Wallac 1420 Victor2).

### Western blot and antibodies

Westen Blot experiments were performed as described [38]. Rabbit polyclonal IgG anti-PPARγ (Santa Cruz Biotechnology, sc-7196), rabbit polyclonal IgG anti-Nrf2 (Santacruz Biotechnology, sc-13032) and goat polyclonal IgG anti-actin (Santacruz biotechnology, sc-1615) were used. Secondary polyclonal anti-goat or -rabbit IgG HRP coupled Abs were used (Cell Signaling).

### Murine malaria models

*In vivo* assays were performed with *Plasmodium berghei*. Parasites were administered intraperitoneally (1.10^6^ parasites/mouse) in Swiss female 12 week-old mice. Studies were performed on separate groups of 7 mice each infected and were repeated twice. Two days before infection, mice were treated subcutaneously with PGN (200 µg/mouse). Mice were then treated by oral route with d,L-sulforaphane (Santacruz Bio) (75 mg/kg), rosiglitazone (3 mg/kg) or with vehicule 5 days following the infection. The mice were then monitored daily for parasitemia levels with thin blood smears and survival was assessed twice daily.

### Statistical analysis

For each *in vitro* experiments, the data were subjected to one-way analysis of variance followed by the means multiple comparison method of Bonferroni-Dunnet. p<0.05 was considered as the level of statistical significance. Survival studies were done using 7 mice per group and were repeated twice. Statistical significance was determined by a log-rank test.

## Supporting Information

Figure S1 **PPARγ activators failed to increase CD36 expression in inflammatory conditions driven by TNF-α or following TLR2 activation.** (A) FACS data showing how cells were gated in the R1 region, a macrophage population double-labeled by the specific murine macrophage marker F4/80 and CD36. (B–C) Representative FACS profiles of CD36 in control macrophages (light grey histogram) and TNF-α (10 ng/mL) or *P.f*. cs-treated macrophages (dark grey histogram) after rosiglitazone (5 µM) or IL-13 (50 ng/mL) stimulations.
(PDF)

Figure S2 **Nrf2 activators promote CD36 and PPARγ expression in inflammatory conditions.** (A–B) Representative FACS profiles of CD36 in control macrophages (light grey histogram) and TNF-α- (10 ng/mL) or *P.f*. cs-treated macrophages (dark grey histogram) after DEM (100 µM) or SFN (10 µM) stimulations. (C) CD36 protein level detected by flow cytometry on Swiss murine peritoneal macrophages firstly incubated during 20 h with rosiglitazone (5 µM), IL13 (50 ng/mL), SFN (10 µM) or DEM (100 µM) and treated during 24 supplementary hours with TNF-α (10 ng/mL). Data are represented as a mean ± SD of three independent experiments. **p<0.01 compared with the respective control (untreated). ^##^p<0.01 compared with the respective control (TNF-α treated cells). (D) CD36 protein level detected by flow cytometry on Swiss murine peritoneal macrophages firstly incubated during 20 h with TNF-α (10 ng/mL) and treated during 24 supplementary hours with rosiglitazone (5 µM) and SFN (100 µM). Data are represented as a mean ± SD of three independent experiments. **p<0.01 and *p<0.05 compared with the respective control (untreated). ^##^p<0.01 compared with the respective control (TNF-α treated cells). (E–F) PPARγ mRNA level on Swiss and C57BL/6 Nrf2^+/+^ and Nrf2^−/−^ murine peritoneal macrophages after treatment with TNF-α (10 ng/mL) during 24 h and then incubation during 5 supplementary hours with SFN (10 µM) or DEM (100 µM). Data are from a representative experiment performed in triplicate ± SD. Experiment has been repeated three times **p<0.01 and *p<0.05 compared with the respective control, ^##^p<0.01 and ^#^p<0.05 compared with the respective control.
(PDF)

Figure S3 **Nrf2 dependent increase of HO-1 mRNA expression on murine macrophages both in normal and in inflammatory conditions.** Nrf2 (A) and HO-1 (B) mRNA expression on Swiss peritoneal macrophages after treatment during 24 h with TNF-α (10 ng/mL) transfected with siRNA targeting Nrf2 (siRNA Nrf2) or control siRNA (siRNA control) and stimulated with sulforaphane (SFN) (10 µM) or diethylmaleate (DEM) (100 µM). Data are represented as a mean ± SD of three independent experiments. *p<0.05 compared with control cells transfected with siRNA control, ^¤^p<0.05 compared with cells transfected with siRNA control and stimulated by SFN. ^§^p<0.05 compared with cells transfected with siRNA control and stimulated by DEM. (C) HO-1 mRNA level on Nrf2+/+ and Nrf2-/- C57BL/6 murine peritoneal macrophages after treatment during 24 h with TNF-α (10 ng/mL) and incubated during 5 supplementary hours with sulforaphane (SFN) (10 µM) or dietylmaleate (DEM) (100 µM). Data are from a representative experiment performed in triplicate ± SD. Experiment has been repeated three times. **p<0.01 compared with the respective control (Nrf2^+/+^), ^##^p<0.01 compared with the respective control (Nrf2^+/+^ cells treated with TNF-α).
(PDF)

Figure S4 **Nrf2 but not PPARγ activators promote CD36 expression on hMDMs inflammatory macrophages.** CD36 protein (A) and CD36 mRNA level (B) on human monocyte-derived macrophages (hMDMs) was quantified by flow cytometry or qRT-PCR experiments after treatment of cells with TNF-α (10 ng/mL), PGN (1 µg/mL) or *P.f.* c s. Data are represented as a mean ± SD of three separate experiments. *p<0.05 and **p<0.01 compared with control cells. (C) FACS data showing how cells were gated in the R1 region, a human macrophage population highly expressing CD36 (D) Representative FACS profiles of CD36 in control hMDMs (light grey histogram) and PGN-treated hMDMs (dark grey histogram) after rosiglitazone (5 µM), SFN (10 µM) or DEM (100 µM) stimulations.
(PDF)

Figure S5 ***In vivo*** **SFN treatment did not alter TNF-α, IFN-γ, IL-12, IL-10 cytokine mRNA levels in macrophages.** (A–B) mRNA levels of Nrf2 target genes and pro- or anti-inflammatory markers on macrophages harvested from 3 days-infected mice treated with PGN and SFN. Data are represented as a mean ± SD of 5 independent mice. ^¤^p<0.05 compared with PGN treated cells.
(PDF)

Table S1 **Murine and human primers sequences used in quantitative PCR experiments.**
(PDF)
