# Supplementary figures and images for: Nrf2, a PPARγ Alternative Pathway to Promote CD36 Expression on Inflammatory Macrophages: Implication for Malaria
Source: PLoS Pathog. 2011 Sep 15;7(9):e1002254. doi: 10.1371/journal.ppat.1002254 (PMC3174257; doi:10.1371/journal.ppat.1002254)

**A**

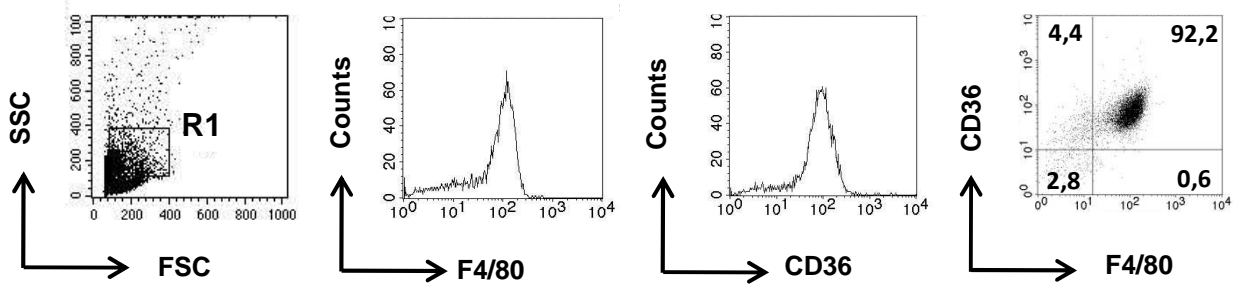

**B**

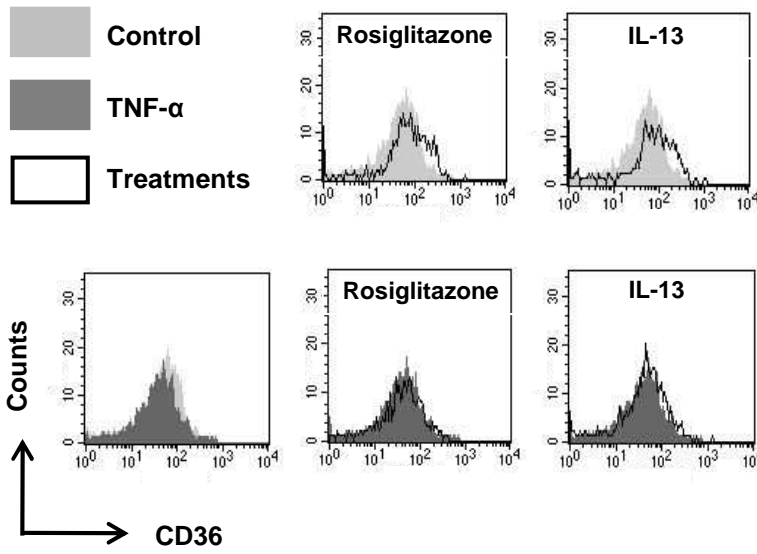

**C**

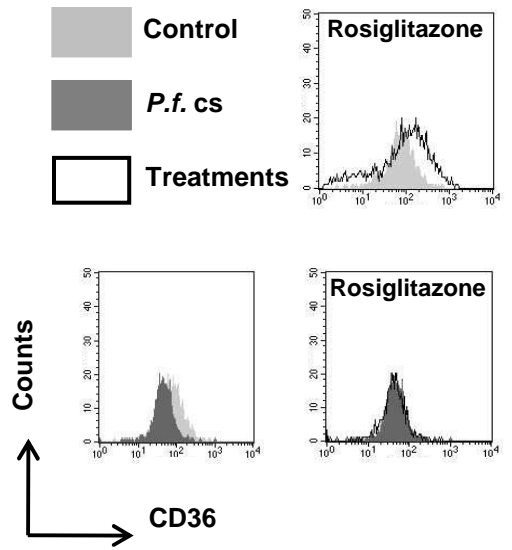

Supplement: Figure S1 — PPARγ activators failed to increase CD36 expression in inflammatory conditions driven by TNF-α or following TLR2 activation. (A) FACS data showing how cells were gated in the R1 region, a macrophage population double-labeled by the specific murine macrophage marker F4/80 and CD36. (B–C) Representative FACS profiles of CD36 in control macrophages (light grey histogram) and TNF-α (10 ng/mL) or P.f. cs-treated macrophages (dark grey histogram) after rosiglitazone (5 µM) or IL-13 (50 ng/mL) stimulations. (PDF) [file ppat.1002254.s001.pdf]

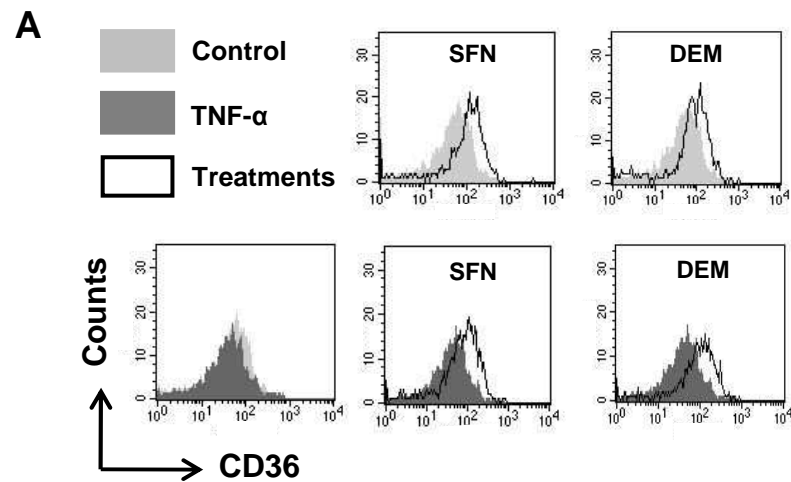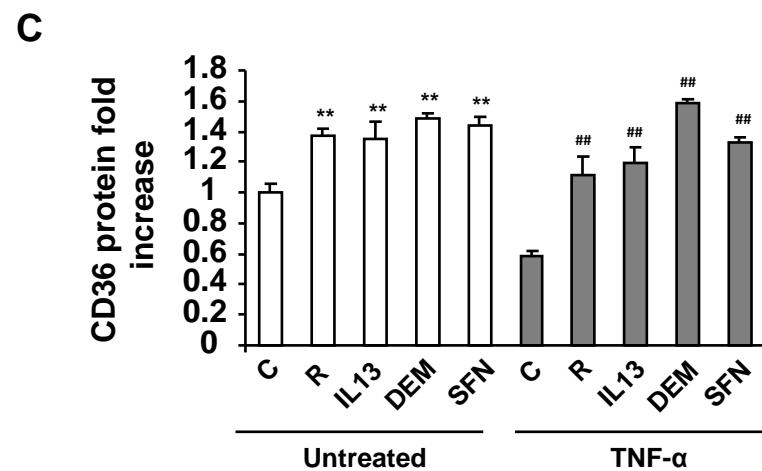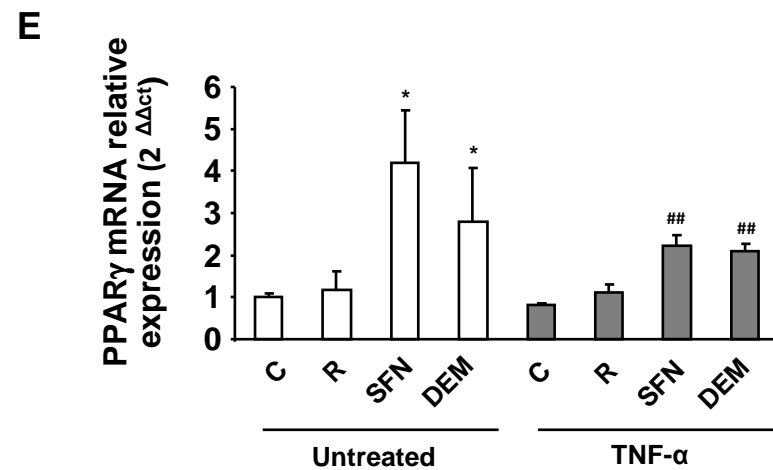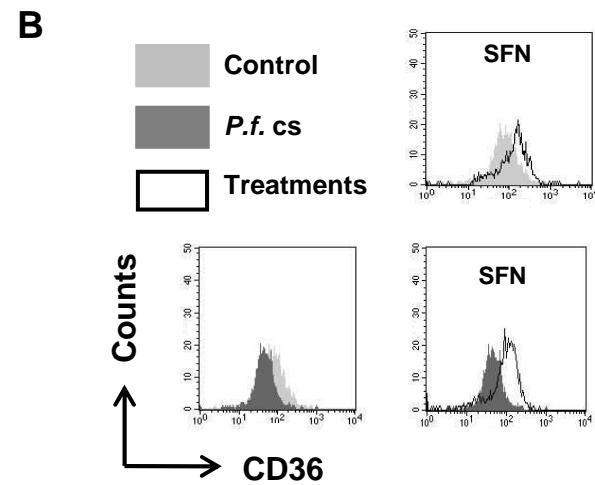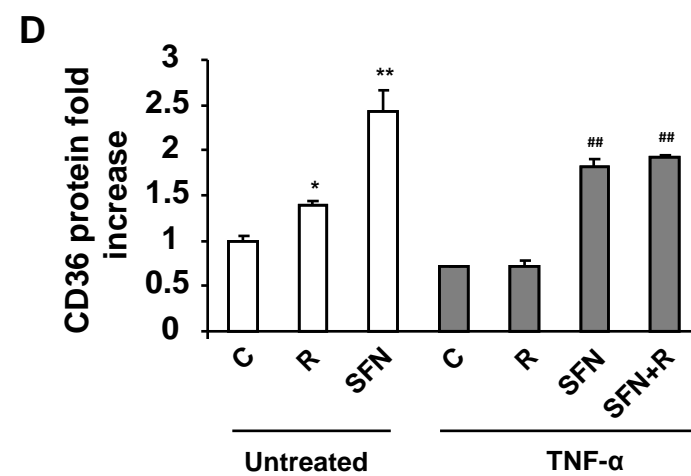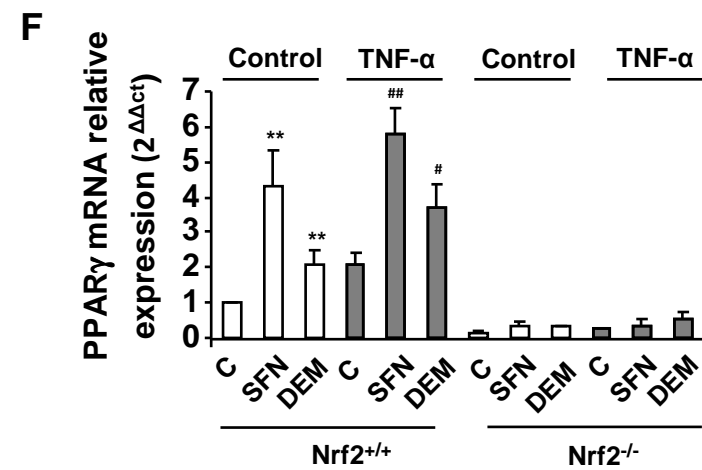

Supplement: Figure S2 — Nrf2 activators promote CD36 and PPARγ expression in inflammatory conditions. (A–B) Representative FACS profiles of CD36 in control macrophages (light grey histogram) and TNF-α- (10 ng/mL) or P.f. cs-treated macrophages (dark grey histogram) after DEM (100 µM) or SFN (10 µM) stimulations. (C) CD36 protein level detected by flow cytometry on Swiss murine peritoneal macrophages firstly incubated during 20 h with rosiglitazone (5 µM), IL13 (50 ng/mL), SFN (10 µM) or DEM (100 µM) and treated during 24 supplementary hours with TNF-α (10 ng/mL). Data are represented as a mean ± SD of three independent experiments. **p<0.01 compared with the respective control (untreated). ##p<0.01 compared with the respective control (TNF-α treated cells). (D) CD36 protein level detected by flow cytometry on Swiss murine peritoneal macrophages firstly incubated during 20 h with TNF-α (10 ng/mL) and treated during 24 supplementary hours with rosiglitazone (5 µM) and SFN (100 µM). Data are represented as a mean ± SD of three independent experiments. **p<0.01 and *p<0.05 compared with the respective control (untreated). ##p<0.01 compared with the respective control (TNF-α treated cells). (E–F) PPARγ mRNA level on Swiss and C57BL/6 Nrf2+/+ and Nrf2−/− murine peritoneal macrophages after treatment with TNF-α (10 ng/mL) during 24 h and then incubation during 5 supplementary hours with SFN (10 µM) or DEM (100 µM). Data are from a representative experiment performed in triplicate ± SD. Experiment has been repeated three times **p<0.01 and *p<0.05 compared with the respective control, ##p<0.01 and #p<0.05 compared with the respective control. (PDF) [file ppat.1002254.s002.pdf]

**A**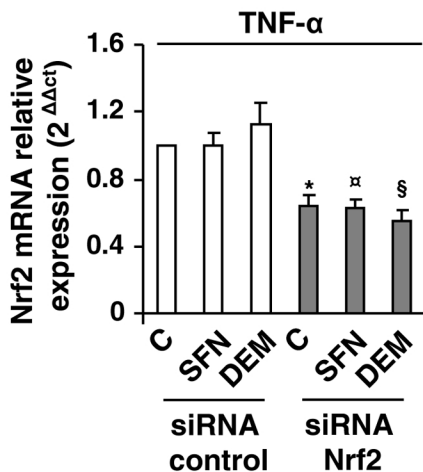**B**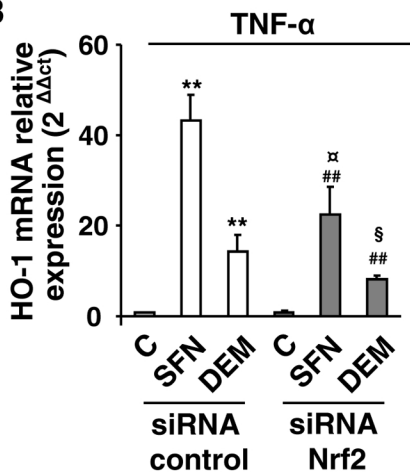**C**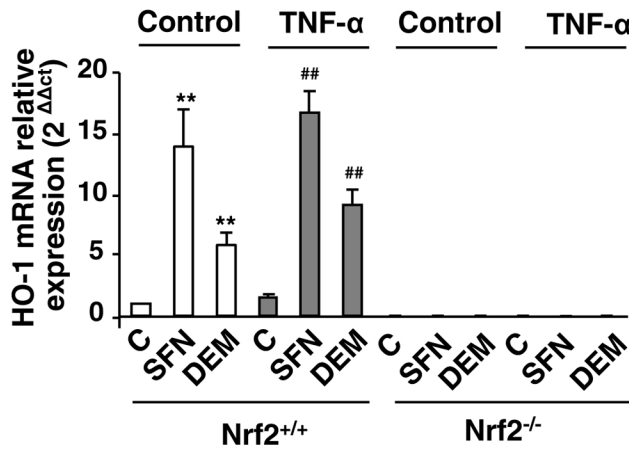

Supplement: Figure S3 — Nrf2 dependent increase of HO-1 mRNA expression on murine macrophages both in normal and in inflammatory conditions. Nrf2 (A) and HO-1 (B) mRNA expression on Swiss peritoneal macrophages after treatment during 24 h with TNF-α (10 ng/mL) transfected with siRNA targeting Nrf2 (siRNA Nrf2) or control siRNA (siRNA control) and stimulated with sulforaphane (SFN) (10 µM) or diethylmaleate (DEM) (100 µM). Data are represented as a mean ± SD of three independent experiments. *p<0.05 compared with control cells transfected with siRNA control, ¤p<0.05 compared with cells transfected with siRNA control and stimulated by SFN. §p<0.05 compared with cells transfected with siRNA control and stimulated by DEM. (C) HO-1 mRNA level on Nrf2+/+ and Nrf2-/- C57BL/6 murine peritoneal macrophages after treatment during 24 h with TNF-α (10 ng/mL) and incubated during 5 supplementary hours with sulforaphane (SFN) (10 µM) or dietylmaleate (DEM) (100 µM). Data are from a representative experiment performed in triplicate ± SD. Experiment has been repeated three times. **p<0.01 compared with the respective control (Nrf2+/+), ##p<0.01 compared with the respective control (Nrf2+/+ cells treated with TNF-α). (PDF) [file ppat.1002254.s003.pdf]

**A**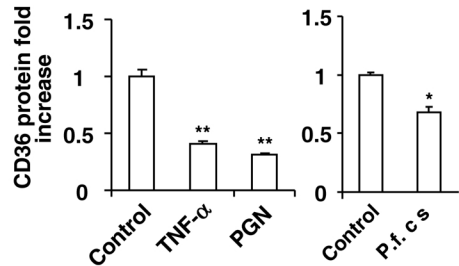**C**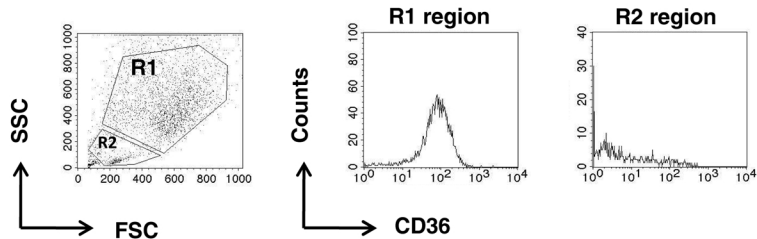**B**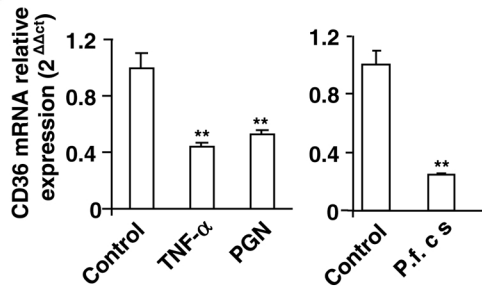**D**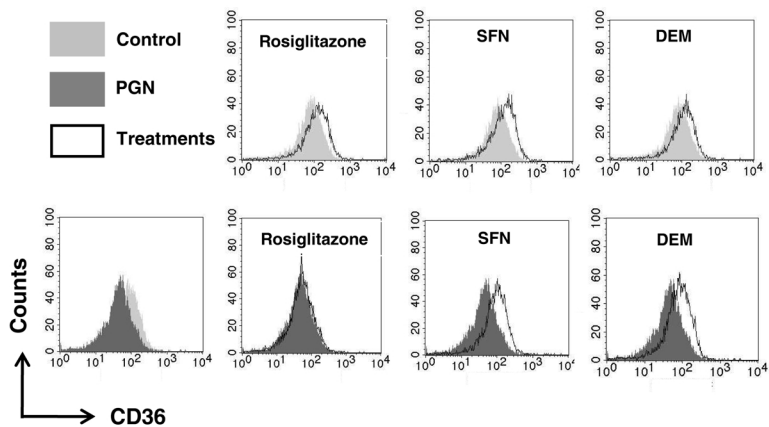

Supplement: Figure S4 — Nrf2 but not PPARγ activators promote CD36 expression on hMDMs inflammatory macrophages. CD36 protein (A) and CD36 mRNA level (B) on human monocyte-derived macrophages (hMDMs) was quantified by flow cytometry or qRT-PCR experiments after treatment of cells with TNF-α (10 ng/mL), PGN (1 µg/mL) or P.f. c s. Data are represented as a mean ± SD of three separate experiments. *p<0.05 and **p<0.01 compared with control cells. (C) FACS data showing how cells were gated in the R1 region, a human macrophage population highly expressing CD36 (D) Representative FACS profiles of CD36 in control hMDMs (light grey histogram) and PGN-treated hMDMs (dark grey histogram) after rosiglitazone (5 µM), SFN (10 µM) or DEM (100 µM) stimulations. (PDF) [file ppat.1002254.s004.pdf]

**A**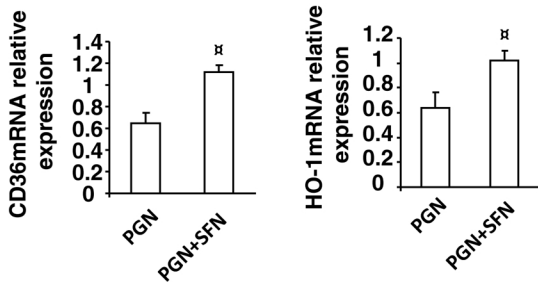**B**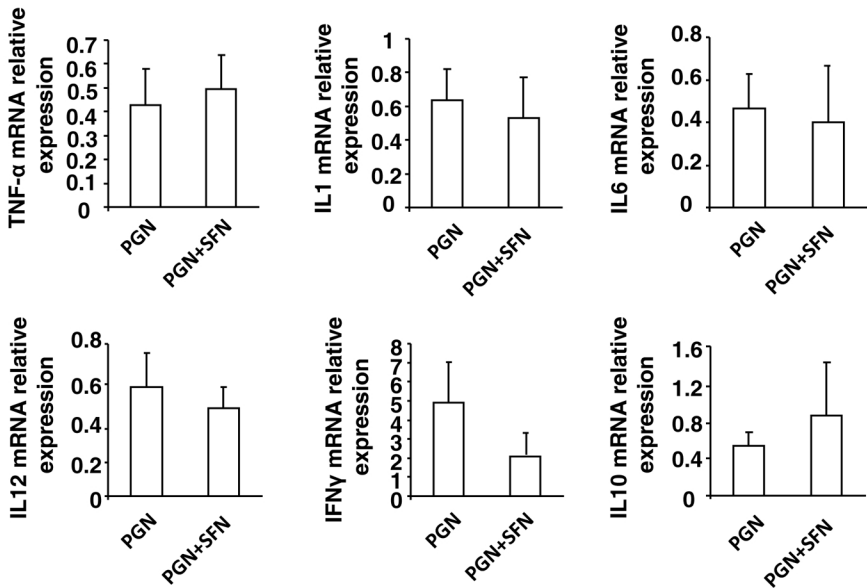

Supplement: Figure S5 — In vivo SFN treatment did not alter TNF-α, IFN-γ, IL-12, IL-10 cytokine mRNA levels in macrophages. (A–B) mRNA levels of Nrf2 target genes and pro- or anti-inflammatory markers on macrophages harvested from 3 days-infected mice treated with PGN and SFN. Data are represented as a mean ± SD of 5 independent mice. ¤p<0.05 compared with PGN treated cells. (PDF) [file ppat.1002254.s005.pdf]
