# Supplementary material for: Nrf2, a PPARγ Alternative Pathway to Promote CD36 Expression on Inflammatory Macrophages: Implication for Malaria
Source: PLoS Pathog. 2011 Sep 15;7(9):e1002254. doi: 10.1371/journal.ppat.1002254 (PMC3174257; doi:10.1371/journal.ppat.1002254)
Supplement: Table S1 — Murine and human primers sequences used in quantitative PCR experiments. (PDF) [file ppat.1002254.s006.pdf]

Murine genes	Sequences
β-actin	sense 5’AGC CAT GTA CGT AGC CAT CC3’<br>antisense 5’CTC TCA GCT GTG GTG GTG AA3’
CD36	sense 5’GAG CAA CTG GTG GAT GGT TT3’<br>antisense 5’GCA GAA TCA AGG GAG AGC AC3’
HO-1	sense 5’CCA GAG TGT TCA TTC GAG CA3<br>antisense 5’CAC GCA TAT ACC CGC TAC CT3’
Nrf2	sense 5’CTC GCT GGA AAA AGA AGT GG-3’<br>antisense 5’CCG TCC AGG AGT TCA GAG AG3’
PPARγ	sense 5’AAT CCT TGG CCC TCT GAG AT3’<br>antisense 5’TTT TCA AGG GTG CCA GTT TC3’
Human genes	Sequences
β-actin	sense 5’CCT CAC CCT GAA GTA CC CA3’<br>antisense 5’TGC CAG ATT TTC TCC ATG TCG3’
CD36	sense 5’TGT AAC CCA GGA CGC TGA GG3’<br>antisense 5’GAA GGT TCG AAG ATG GCA CC3’
PPARγ	sense 5’TTG TCA CGG AAC ACG TGC A3’<br>antisense 5’GGA GCG GGT GAA GAC TCA TG3’
